# Influence of a Physical Exercise Program in the Anxiety and Depression in Children with Obesity

**DOI:** 10.3390/ijerph17134655

**Published:** 2020-06-28

**Authors:** Ena Monserrat Romero-Pérez, Jerónimo J González-Bernal, Raúl Soto-Cámara, Josefa González-Santos, José Manuel Tánori-Tapia, Paula Rodríguez-Fernández, María Jiménez-Barrios, Sara Márquez, José Antonio de Paz

**Affiliations:** 1 Department of Sports and Physical Activity Sciences, University of Sonora, Sonora 83067, Mexico; ena.romero@unison.mx; 2 Department of Health Sciences, University of Burgos, 09001 Burgos, Spain; mjgonzalez@ubu.es (J.G.-S.); prf0011@alu.ubu.es (P.R.-F.); mjb0007@alu.ubu.es (M.J.-B.); 3 Institute of Biomedicine, University of León, 24071 León, Spain; sara.marquez@unileon.es (S.M.); japazf@unileon.es (J.A.d.P.)

**Keywords:** obesity, children, physical exercise, anxiety, depression

## Abstract

(1) *Background*: The high prevalence of childhood obesity and its multicausal etiology make it necessary to approach it through different strategies, whose objective is to promote the physical, mental, and social well-being of children. Regular physical activity, in addition to having positive effects on the physical environment of those who practice it, influences positively in psychological aspects such as anxiety and depression, which are very frequent in children with obesity and overweight. (2) *Objective*: To analyze the changes produced by a program of physical exercise based on anthropometric indicators and levels of anxiety and depression in a population of Mexican children with obesity. (3) *Methods*: A longitudinal study with experimental group (EG) and control group (CG). The analysis population consisted of 105 children with a body mass index (BMI) for their gender and age group above the 95th percentile, of which 60 were girls and 45 were boys, with a mean age of 10.02 years (SD ± 0.79). By randomizing the participants, 54 were part of the EG and 51 of the, CG The EG participated in a physical exercise program, distributed in two weekly sessions, each lasting 50 min, for 20 consecutive weeks. The CG group continued its usual activities during the intervention period. An inferential analysis was performed between the socio-demographic, anthropometric and psychological variables. (4) *Results*: The implementation of a physical exercise program in children with obesity favors the appearance of positive thoughts, with improvements in their emotional well-being, self-perception and self-concept; although it does not produce significant changes in weight, height, Z-Score, level of anxiety or depressive thoughts. (5) *Discussion*: Regular physical exercise practice has positive effects on mental health, although new studies are required to analyze specifically its influence on anxiety and depression in children with obesity.

## 1. Introduction

Globally, childhood obesity has become one of the major public health problems, due to its serious consequences on the physical, psychiatric, and psychosocial environment of children. It has a multicausal etiology, in which factors such as genetic predisposition, breastfeeding, family environment, basal metabolic rate, and healthy lifestyle habits in terms of physical activity and diet stand out [1,2].

According to the World Health Organization (WHO) [3], in the last 40 years, the number of children and adolescents who have suffered from obesity worldwide has multiplied by 10, reaching 50 million girls and 74 million children in 2016 [4]. In Mexico, the latest available data from the Health and Nutrition Survey estimates a prevalence of obesity at school age of 33.2%, being higher in boys than girls (33.7% versus 32.8%) [5]. This increasing evolution of the figures may probably be due to high caloric intake and poor nutritional quality with an excess consumption of carbohydrates and saturated fatty acids, characteristics of ultra-processed foods, as well as a decrease in physical activity [6,7].

Children with obesity often suffer rejection from their peers. This rejection can trigger antisocial attitudes, depression, anxiety or inactivity, which can favor an increase in food intake, in turn aggravating this pathology [8,9,10]. Compared to children with normal weight, those with obesity are more likely to have high levels of psychosocial distress, directly related to depressive symptoms and anxiety [1,11,12].

The possible impact that all these aspects can have on school performance in children has also been studied and, although the conclusions are not consistent, it is an aspect that should be considered in the long-term follow-up of these children [13,14].

The benefits of children’s physical activity on the health of the heart, bones and muscles, as well as its effects on the prevention of type 2 diabetes mellitus and obesity, have been extensively studied [15]. Physical activity, in addition to being essential for addressing the physical symptoms of obesity, has also been recommended in promoting mental health and as a complementary treatment for mental illness, especially for children that experience stigma based on their weight. Physical activity provides opportunities for fun, socializing, reaching goals and developing relationships [16,17]. The benefits of its regular practice are amplified when it is started at an early age, avoiding the development of diseases associated with a sedentary lifestyle [18].

The promotion of physical activity and the reduction of sedentary behaviors could protect the mental health of children and adolescents. A sedentary lifestyle is associated with greater psychological distress such as depression and anxiety, and less psychological well-being, life satisfaction, and happiness [18,19,20,21,22,23]. Specifically, it has been shown that depressive symptoms in children with obesity or overweight are directly related to the time they maintain sedentary behaviors, such as being in front of a computer screen or a mobile device [24].

Despite the limited evidence available on the influence of physical activity on childhood depression and anxiety, especially in obese children, it has been shown that those who practice physical activity regularly have better self-perception and greater self-esteem. This situation will lead to greater satisfaction with life and, consequently, a decrease in symptoms related to anxiety and depression [25,26]. Likewise, health promotion strategies aimed at promoting healthy lifestyle habits, such as the habitual practice of physical exercise, could benefit children’s self-esteem as well as the mental health and quality of life of children as they develop into adults [25].

However, despite the increasing number of investigations that suggest that physical exercise promotes better mental health in children and adolescents, the recommended intensity and frequency have not yet been determined [27,28,29].

Due to the important role of physical exercise in the prevention and management of psychological disorders in children with obesity or overweight as well as the limited scientific evidence available on its effects, the present study was proposed, whose objective was to analyze the changes produced by a program of physical exercise based on anthropometric indicators and levels of anxiety and depression in a population of Mexican children with obesity. Therefore, the research question was whether a program of physical exercise was effective in the improvement of depression or anxiety in children with obesity.

## 2. Materials and Methods

### 2.1. Study Design—Participants

The sample consisted of 105 schoolchildren, 60 girls and 45 boys, with a mean age of 10.02 years (SD ± 0.79). Participants between 8 and 9 years old formed 54.29% of the total, while the remaining 45.71% were between 10 and 11 years old. Participants were stratified by age and gender and randomly allocated to the experimental group (EG) or control group (CG), using the Epidat version 4.2 program. This is a freely distributed epidemiological and statistical data analysis software, which facilitates the randomization process of subjects included in studies. It has been developed by the Ministry of Health (Spain) with the support of the Pan American Health Organization (PAHO-WHO) and the CES University of Colombia. Of the 105 children, 54 were part of the EG and 51 of the, CG During the development of the study, the presence of side effects was not observed, nor did any loss of participants occur.

A longitudinal study, with experimental group (EG) and control group (CG), was performed. Its study population was all primary school students, aged between 8 and 11 years, with a body mass index (BMI) for their gender and age group higher than the 95th percentile, according to the 2007 WHO growth references data [30], who attended 3 of the 343 schools in primary education in the State of Sonora (Mexico), which were randomly selected.

Those students who had some type of physical disability, which made it impossible to carry out the different activities or an intellectual disability, which made it difficult to understand the instructions, as well as those who suffered from chronic diseases such as hypothyroidism or type 1 diabetes mellitus were excluded.

### 2.2. Procedure

Prior to starting the study, parents of possible participants were contacted. Different meetings were held, in which the objective and methodology of the study as well as its voluntary nature were explained to them. They were invited to participate in the study, having to sign the informed consent in case of acceptance.

After verifying compliance with the established inclusion criteria, students were selected by a non-probability convenience sampling. The baseline assessment of all participants was carried out by the same evaluators and on successive days, following this chronological order: a first session aimed at obtaining data referring to the clinical history, history of psychological development and socio-demographic profile of the child as well as the performance of anthropometric tests; and two sessions for the administration of psychological tests. The approximate duration of each of them was 20–25 min per child. These sessions were held at school, in a classroom with good lighting, ventilation, and free from distractions. This same assessment was repeated for all students after a follow-up period of 20 weeks.

School children included in the EG participated in a physical activity program in which they received two weekly 50-min sessions, for 20 consecutive weeks. All sessions were structured in three fundamental parts: an initial 5-min warm-up, a 40-min core session in which specific exercises and pre-sport games were performed to work on the development of conditional and coordinative capacities, and a final 5 min of relaxation and stretching. In these sessions, through playful activities and mainly aerobic exercise, the children exercised their conditional and coordinative capacities (strength, resistance, coordination, and speed). These sessions were performed in groups, outside school hours, as an extracurricular activity, avoiding the days when the children had their compulsory physical education classes, according to their annual school calendar. The CG was evaluated under the same parameters and at the same time points as the, EG; with the only difference being that they did not participate in any scheduled exercise intervention. During the intervention period, the CG participants continued with their usual activities at the end of the classes.

The study received a favorable report from the Bioethics Committee of the Faculty of Medicine of the Sonora University (Reference DMCS/CBIDMCS/D21), respecting at all times the ethical principles contained in the Helsinki Declaration.

### 2.3. Main Outcomes—Instruments

The anxiety and depression levels of the participating students were the main outcomes from the study. To assess the level and nature of anxiety, the Manifest Anxiety Scale in Children-Revised (CMAS-R), designed by Reynolds and Richmond in 1997 [31], and validated to Spanish language by Gussinyé in 2005 [32], was used. It is a self-administered questionnaire, aimed at children and adolescents between 6 and 18 years old, composed of 37 items and four subscales: physiological anxiety, restlessness, social concerns, and lies. The responses are dichotomous, yes or no, and the sum of the positive answers reflects the total level of anxiety. The internal consistency of the test in its Spanish adaption is 0.83. For the assessment of global and specific depressive symptoms, the Depression Scale in Children (CDS) was used, designed by Lang and Tisher in 1983 [33], adapted to Spanish language by Seisdedos in 2003 [34] and validated in the Sonoran population by Robles in 2008 [35]. It consists of a self-report questionnaire, directed at children between 8 and 16 years old, composed of 66 statements, 48 of them of a depressive type and 18 of a positive type. Each item must be answered by the child using a Likert scale of five points, according to the degree of agreement with the content of the sentence, where 1 means strongly disagree and 5 strongly agree. It is made up of two independent subscales: total depressive (affective response, social problems, self-esteem, concern for death and health, feelings of guilt, and various depressants) and total positive (mood—joy and various positives). The internal consistency of the test in its Spanish adaptation is 0.88. Other data collected were age, gender, weight, height, and body mass index (BMI). For the anthropometric and body composition assessment of the children, a digital column scale SECA model 763, Hamburg, Germany (weight) and a Holtain Limit stadiometer (height) were used. BMI was calculated using the formula: [Weight in kilograms/(Height in meters)^2^], finding the percentiles from the WHO growth tables [36]. The BMI Z-Score value was obtained from the result of the following equation: [Real anthropometric value − median (P50)/Standard deviation (SD)].

### 2.4. Statistical Analysis

Mean and standard deviation (SD) were used for the description of quantitative variables while frequency distribution and percentages were used in the case of categorical variables. The compliance of the normality criteria of the quantitative variables was assessed by Kolmogorov–Smirnov test. In cases where the normal distribution could not be assumed, median and interquartile range (IQR) were calculated. For the analysis of changes in the level of anxiety and depression between the CG and, EG; at different time points, the Mann–Whitney U test was used while the Student’s t test was used for independent samples for the analysis of changes in the anthropometric variables between both groups. Spearman correlation was used to compare the differential scores obtained in the anthropometric and psychological variables. For the analysis of statistical significance, a value of *p* < 0.05 was established. Statistical analysis was performed with SPSS version 25 software (IBM-Inc, Chicago, IL, USA).

## 3. Results

Of the 105 students who participated in the study, 51 were assigned to the GC and the remaining 54 to the EG. The percentage of boys included in the EG was higher than in the CG (41.18% versus 44.44%), while the number of girls included in both groups was the same (*n* = 30). The mean age of schoolchildren was significantly higher in the EG (10.28 ± 0.96 years versus 9.47 ± 0.40 years; *p* < 0.001), with no significant differences regarding gender. At the beginning of the study, the children’s weight, height, and BMI *Z*-Score mean were 52.70 kg (SD ± 10.91), 140.57 cm (SD ± 8.43), and 2.88 (SD ± 0.72), respectively; obtaining the following values after 20 weeks, 53.62 kg (SD ± 10.90), 142.33 cm (SD ± 8.50), and 2.82 (SD ± 0.71). The median score obtained in the psychological aspects by the students at the start and at 20 weeks were very similar: 19.00 (IQR 15.00–22.00) versus 19.00 (IQR 15.00–23.00) for anxiety and 143.00 (IQR 121.00–173.00) versus 142.00 (IQR 122.00–177.00) for depression. The values obtained in the anthropometric and psychological variables of obese children, according to the type of group in which they were included, before and after performing the physical activity intervention are summarized in Table 1 and Table 2.

When comparing the differential scores of the anthropometric and psychological variables, after a follow-up period of 20 weeks, no statistically significant differences were observed between the CG and the EG in weight (t_(103)_ = −0.194; *p* = 0.466), anxiety level (U = 1440.50; *p* = 0.683) or depressive thoughts (U = 1399.50; *p* = 0.885). However, the implemented physical activity program in children with obesity had an effect on height (t_(103)_ = 1.782; *p* = 0.019), the BMI Z-Score (t_(103)_ = −1.074; *p* = 0.007) and the development of positive thoughts such as joy (U = 950.50; *p* = 0.006). (Table 3 and Table 4).

Changes in weight, height or BMI *Z*-Score, after a follow-up period of 20 weeks, were not correlated with the differential scores obtained in the psychological variables, both in the children included in the CG and in the, EG However, the improvement in positive thoughts was correlated with a lower number of depressive thoughts in children with obesity (r_(105)_ = −206; *p* = 0.020).

## 4. Discussion

In this study, the effects of a physical exercise program on the mental health of children with obesity have been analyzed, studying its possible benefits on anxiety and depression.

Regular physical exercise has been shown to be an effective strategy for improving the clinical situation of children and adolescents diagnosed with depression [8,27,28,29,37]. However, despite the growing number of studies linking physical activity with reduced anxiety, both in the general population and in those with some type of disorder [38], its specific effects on overweight or obese schoolchildren have not been determined.

In a recent study, based on a four-week program of physical activity, targeting overweight 12–14 year-olds, the body weight and BMI values have been found to be significantly different between the pre- and post-intervention tests, without reference to psychological variables [39]. In contrast with these results, there were no improvements in the anthropometric variables in this study, which may be related to the longer duration of the physical activity intervention.

In this study, the regular practice of physical activity promotes the appearance of positive thoughts about oneself, increases self-esteem, and improves emotional well-being and self-concept, decreasing the appearance of negative thoughts. In a meta-analysis, whose objective was to investigate the effect of practicing physical activity on the prevention of depression in children and adolescents, the results allow the conclusion that a higher level of physical activity is associated with a decrease in depressive symptoms, highlighting that the number of systematic reviews carried out at an early age is very low. Furthermore, the fact that most of the studies related to this field are cross-sectional limits the ability to investigate their causality, indicating an inverse association between physical activity and depressive symptoms [40].

Physical activity promotes physical and mental health, improves the mood and increases life satisfaction, which indirectly reduces symptoms related to anxiety and depression [41,42,43,44,45,46,47,48,49]. In this study, positive thoughts, such as joy, contribute to greater satisfaction with life and, in the same way, reduce depressive symptoms. In a systematic review carried out by Ruotsalainen et al., in which the effects of physical activity on certain psychological aspects in overweight children and adolescents was evaluated, it is concluded that this type of intervention improves self-perception and body satisfaction, but not the symptoms of depression [41]. This conclusion is similar to those obtained in this study when it was affirmed that improvements in depressive thoughts increase positive thoughts, independently of the intervention itself.

The mental health of Mexican schoolchildren with obesity has improved in aspects related to self-esteem, self-perception, and self-concept. Lubans et al. have shown that, although the mechanisms by which physical exercise influences cognitive and mental health have not been yet established, its regular practice has a significant impact on mental health, specifically in self-perception and, in consequence, in other variables, such as self-esteem [43]. For their part, Whooten et al., after carrying out a program of physical activity for 12 weeks in children older than 8 years with obesity, have shown that those who participated in the program, in addition to losing weight, experienced improvements in social and emotional well-being, without express reference to specific psychological aspects [48].

The study findings must be considered within the context of their strengths and limitations. Among their strengths are the collection of data in three different schools in the State of Sonora (Mexico) and its longitudinal design, which allows determining the existence of causal relationships and overcoming the limitations found in previous cross-sectional studies that addressed this issue. Although the effects of educational intervention on different psychological variables have been analyzed using validated and reliable instruments, the fact that many of them are not specifically intended to assess these aspects in the child population with obesity problems can be considered as a limitation, making it difficult to obtain more detailed data.

## 5. Conclusions

The implementation of a physical exercise program, aimed at children with obesity, has not been related to changes in their anthropometric variables (weight, height, *Z*-Score) or in their levels of anxiety or depressive thoughts. However, regular practice of physical activity induces the appearance of positive thoughts about themselves, increasing self-esteem and improving emotional well-being and self-concept, while reducing the appearance of depressive thoughts.

Taking into account these results and the small number of studies evaluating the effects of physical exercise on different psychological aspects in children with obesity, the development of new research on this topic is justified, with representative samples and at an early age.

## Figures and Tables

**Table 1:** Values of anthropometric variables according to the type of group, before and after of performing the physical activity intervention.

	Group	Pre-Intervention		Post-Intervention	
		Mean	SD	Mean	SD
**Weight**	CG	47.90	7.09	48.74	7.25
	EG	52.24	11.96	58.23	11.78
**Height**	CG	136.26	6.44	138.41	6.70
	EG	144.64	8.10	146.03	8.42
**BMI *Z*-Score**	CG	2.80	0.55	2.63	0.52
	EG	2.96	0.85	2.86	0.84

CG: Control group; EG: Experimental group; SD: Standard deviation.

**Table 2:** Values of psychological variables according to the type of group, before and after performing the physical activity intervention.

	Group	Pre-Intervention		Post-Intervention	
		Median	IQR	Median	IQR
**Anxiety**	CG	20.00	16.00–22.00	19.00	16.00–23.00
	EG	18.00	13.50–22.25	19.00	14.75–22.25
**Total positives**	CG	1338.00	121.00–173.00	136.00	122.00–173.00
	EG	147.00	123.75–176.00	144.00	121.75–173.50
**Total depressive**	CG	39.00	30.00–49.00	39.00	33.00–48.00
	EG	42.00	36.50–46.00	41.00	31.75–46.25

Control group; EG: Experimental group; IQR: Interquartile range.

**Table 3:** Comparison of differential scores of anthropometric variables according to the type of group.

	Group	Mean	SD	Student´s T	*p*-Value
**DS Weight**	CG	0.84	0.92	−0.19	0.466
	EG	0.99	5.51		
**DS Height**	CG	2.16	2.05	1.99	0.019
	EG	1.39	2.33		
**DS BMI *Z*-Score**	CG	−0.40	0.70	2.69	0.007
	EG	0.02	2.71		

DS: Differential score; BMI: Body mass index; CG: Control group; EG: Experimental group; SD: Standard deviation.

**Table 4:** Comparison of differential scores of psychological variables according to the type of group.

	Group	Median	IQR	Mann–Whitney U	*p*-Value
**DS Anxiety**	CG	0.00	0.00–1.00	1440.50	0.683
	EG	3.00	7.00–5.25		
**DS Total positives**	CG	0.00	−2.00–3.00	950.50	0.006
	EG	1.50	−3.25–3.00		
**DS Total depressive**	CG	0.00	−3.00–3.00	1399.50	0.885
	EG	1.50	−12.25–15.00		

DS: Differential score; CG: Control group; EG: Experimental group; IQR: Interquartile range.
